# Differential regulation of IFNα, IFNβ and IFNε gene expression in human cervical epithelial cells

**DOI:** 10.1186/s13578-017-0185-z

**Published:** 2017-11-02

**Authors:** Jennifer Couret, Carley Tasker, Jaeha Kim, Tiina Sihvonen, Saahil Fruitwala, Alison J. Quayle, Pierre Lespinasse, Debra S. Heller, Theresa L. Chang

**Affiliations:** 1 Public Health Research Institute, New Jersey Medical School, Rutgers, The State University of New Jersey, 225 Warren Street, Newark, NJ 07103 USA; 2 Department of Microbiology and Molecular Genetics, New Jersey Medical School, Rutgers, The State University of New Jersey, 225 Warren Street, Newark, NJ 07103 USA; 3 Department of Obstetrics, Gynecology & Women’s Health, New Jersey Medical School, Rutgers, The State University of New Jersey, 185 South Orange Ave, Newark, NJ 07101 USA; 4 Department of Pathology & Laboratory Medicine, New Jersey Medical School, Rutgers, The State University of New Jersey, 185 South Orange Ave, Newark, NJ 07101 USA; 5 Department of Microbiology, Immunology and Parasitology, Louisiana State University Health Science Center, New Orleans, LA USA

## Abstract

Interferonε (IFNε) is a unique type I IFN that has distinct functions from IFNα/β. IFNε is constitutively expressed at mucosal tissues, including the female genital mucosa, and is reported to be modulated by estrogen and seminal plasma. However, its regulation by cytokines, including TNFα, IL-1β, IL-6, IL-8, IL-17, IL-22 and IFNα, which are commonly present in the female genital mucosa, is not well documented in freshly isolated primary cervical cells from tissues. We determined the effect of these cytokines on gene expression of type I IFNs in an immortalized endocervical epithelial cell line (A2EN) and in primary cervical epithelial cells. Several pro-inflammatory cytokines were found to induce IFNε, and TNFα induced the strongest response in both cell types. Pretreatment of cells with the IκB inhibitor, which blocks the NF-κB pathway, suppressed TNFα-mediated IFNε gene induction and promoter activation. Expression of IFNα, IFNβ, and IFNε was differentially regulated in response to various cytokines. Taken together, our results show that regulation of these IFNs depends on cell type, cytokine concentration, and incubation time, highlighting the complexity of the cytokine network in the cervical epithelium.

Dear Editor,

Interferonε (IFNε) is a unique, functionally distinct type I IFN [1, 2]. Unlike IFNα/β, whose expression is nearly undetectable at baseline, IFNε is expressed at basal constitutive levels in mucosal tissues, including the female genital mucosa. IFNε gene expression is not induced in response to activation of several Toll-like receptors, nor is it activated by IRF3, IRF5 or IRF7 [3]. While the in vitro anti-viral activity of IFNε is weaker than that of IFNα [4–6], IFNε efficiently clears viral infections in vivo by recruiting lymphocytes and by increasing the numbers and functions of cytotoxic T cell subsets in mucosal tissues [1]. In IFNε−/− mice, reduced NK cell numbers in the female genital tissues are associated with increased susceptibility to HSV2 and chlamydial infection [3]. Of considerable relevance, HIV-negative sex workers with frequent exposure to semen have an increased level of IFNε gene expression in the cervical epithelium and increased numbers of immune effectors cells in their cervical tissues [7], suggesting that IFNε has a protective role in reducing HIV susceptibility by modulating the mucosal immune response.

Expression of IFNε is reported to be regulated by estrogen and seminal plasma [3, 7, 8]; however, the effect of individual cytokines on IFNε expression is less well documented. Matsumiya and colleagues demonstrated that TNFα at a concentration of 10 ng/ml or higher induces IFNε mRNA expression by near twofold in HeLa cells, a cervical cancer cell line, although TNFα did not induce the IFNε promoter activity in this study [9]. Interestingly, the expression of other type I IFNs including IFNα, IFNβ, IFNκ and IFNω, is not induced by TNFα in HeLa cells [9]. In this study, we examined the effect of cytokines commonly found in the mucosa during either a healthy or an inflammatory state, on IFNε expression in A2EN cells, an immortalized endocervical cell line with limited passages [10], and in primary cervical epithelial cells (CECs) derived from various donors. Note that the protein levels were not assessed in this study because there is no reliable ELISA kit for IFNε. Finally, we also determined the involvement of the NF-κB pathway in TNFα-mediated IFNε gene induction and promoter activation.

## Results and discussion

**Fig. 1:**
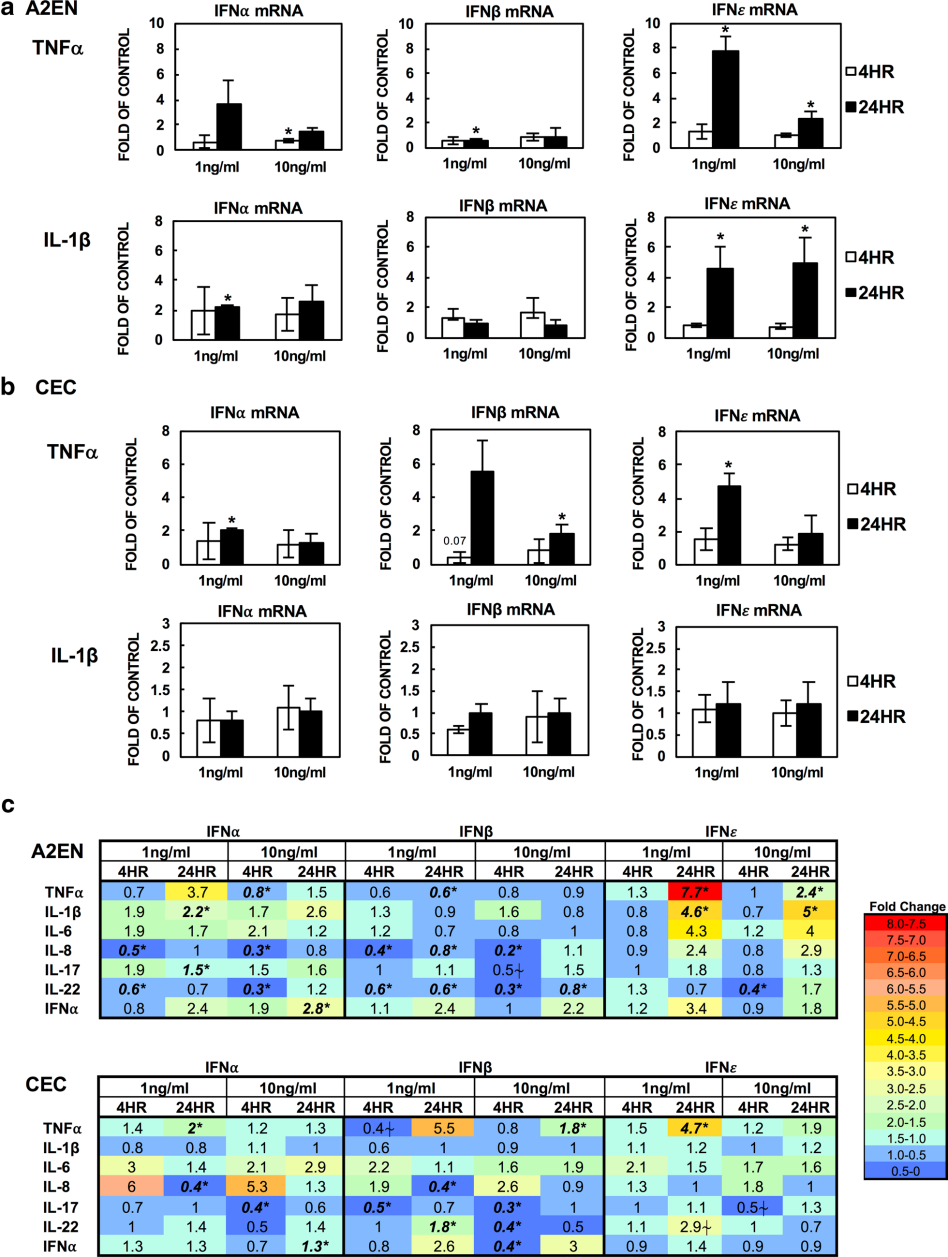
Regulation of IFNα, IFNβ and IFNε by various cytokines in A2EN cells and primary cervical cells. A2EN or primary cervical epithelial cells were treated with stated cytokines at 1 or 10 ng/ml for 4 or 24 h. Total RNAs were prepared and gene expression of IFN⍺, IFNβ and IFNε was measured by real-time RT-PCR. Induction of each gene (as fold-change relative to untreated control) was calculated using the ΔΔCT method as described in “Materials and methods”. The value of 1 indicates the level of gene expression of untreated control cells. Data for TNFα and IL-1β are mean ± SD of four independent experiments in A2EN cells (**a**) and of experiments from primary cervical cells from different donors (**b**). Average of fold-changes of other cytokines-treated samples relative to untreated samples from four different experiments were summarized in **c**. Numbers in bold represents significant changes (*p < 0.05). ⍭p=0.057–0.07

A2EN cells or CECs were treated with stated cytokines at 1 or 10 ng/ml for 4 or 24 h before RNA extraction. Gene expression of IFNα, IFNβ, or IFNε was determined by RT-PCR analysis. As expected, the baseline of IFNα or IFNβ was low in A2EN and CECs. The IFNε mRNA level was 315- and 23-fold more abundant than IFNα and IFNβ mRNAs in A2EN cells, respectively, and 97-fold and 26-fold more abundant than IFNα and IFNβ mRNA levels in CECs, respectively (data not shown). Pro-inflammatory cytokines such as TNFα (1 ng/ml) and IL-1β at 1 and 10 ng/ml significantly induced IFNε by 7.7 and 4.6-fold, respectively. IL-1β had a moderate effect on IFNα expression (twofold induction) in A2EN cells (Fig. 1a). Gene induction of IFNα and IFNε by TNFα was also found in CECs (Fig. 1b). Interestingly, TNFα suppressed IFNβ expression by 40–50% in A2EN cells. IFNβ suppression by TNFα at 1 ng/ml for 4 h incubation in CECs was observed; however, treatment of TNFα for 24 h induced IFNβ in CECs.

IL-6 or IL-8, which are often elevated during inflammation at the mucosa, was noted to induce IFNε in A2EN cells after 24 h incubation, but the induction was not significant when various experiments were compared (Fig. 1c). The effects of IL-6 and IL-8 on IFNε induction were not prominent in CECs. Interestingly, IL-8 significantly suppressed IFNβ in A2EN cells (25–77% suppression) and CECs (61% suppression). IL-8 treatment for 4 h also suppressed IFNα in A2EN cells (53–71% suppression) but the significant suppression of IFNα was observed in CECs after the 1 ng/ml for 24 h incubation (64% suppression).

IL-17 and IL-22 play a crucial role in mucosal immune homeostasis [11]. In A2EN cells, IL-17 slightly elevated the IFNα mRNA level by 1.8-fold but had a trend to suppress expression of IFNβ (p=0.057) (Fig. 1c). In CECs, IL-17 significantly suppressed IFNα and β after 4 h incubation. The suppression of IFNε by IL-17 was observed but not significant (p=0.07). IL-22 has similar but distinct functions compared to IL-17 [11]. In A2EN cells, IL-22 significantly suppressed IFNα and β at both concentrations after the 4 h incubation. IL-22 continued to suppress IFNβ after incubation for 24 h. The suppressive effect of IL-22 on IFNε was limited at 10 ng/ml for 4 h incubation. IL-22 did not have a significant impact on IFNα expression but slightly induced IFNβ or IFNε (1 ng/ml for 24 h) in CECs. IL-22 at 10 ng/ml significantly suppressed IFNβ after 4 h incubation, indicating that IL-22-mediated IFNβ gene regulation depended on the cytokine concentration and the incubation time.

IFNα, induced in response to infection or Toll-like receptor activation, is known to induce type I IFNs [12]. Because type I IFN signaling can be cell-type specific [13], we determined the effect of IFNα on expression of IFNs in A2EN cells and CECs. IFNα moderately induced all three IFNs by 2–3 fold in A2EN cells after 24 h treatment although only induction of IFNα was statistically significant (Fig. 1c). IFNα slightly induced IFNα but had no effect on IFNε in CECs. Regulation of IFNβ by IFNα appeared to be time and concentration dependent. IFNα at 10 ng/ml suppressed IFNβ expression by 60% after 4 h incubation, but there was a trend of IFNβ induction after 24 h incubation.

**Fig. 2:**
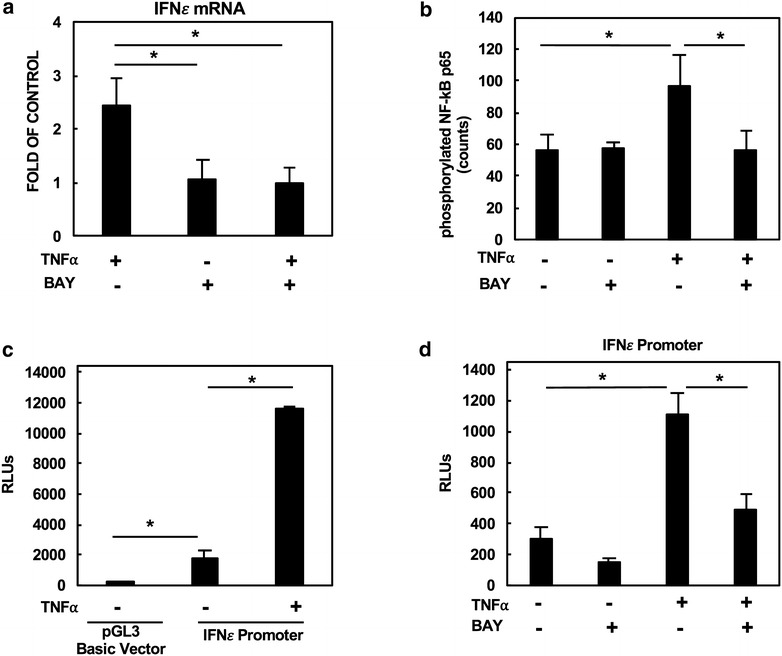
TNFα-mediated IFNε gene expression is mediated by the NFκB pathway. **a** Primary cervical cells were pre-treated with or without IKK inhibitor, BAY 11-7082 at 10μM, for 1 h followed by TNFα (1 ng/ml) stimulation. Total RNA was prepared and IFNε gene expression was measured by RT-qPCR. **b** Primary cervical cells were pre-treated with or without BAY 11-7082 at 10μM for 1 h before TNFα stimulation for 30 min. The Ser 536 phosphorylation of NFκB p65 was measured by Perkin Elmer AlphaLISA SureFire Ultra pNFκB p65 (Ser 536) assay kit according to the manufacture’s instruction. **c** A2EN cells were transfected with pGL3 or pGL3 containing IFNε promoter. The promoterless pGL3 basic vector was included as the baseline control. Cells were then treated with or without 1 ng/ml TNF for 24 h before measuring luciferase activity (RLUs). **d** A2EN cells were transfected with the IFNε promoter construct and then treated with or without BAY 11-7082 at 10μM for 1 h. Cells were then stimulated with or without TNFα at 1 ng/ml for 24 h before the measurement of the luciferase activity. Data are mean ± SD of three independent experiments. *p < 0.05

TNFα is known to activate the transcription factor NFκB [14] although the previous report showed that it did not induce the IFNε promoter activity in HeLa cells [9]. To determine whether the NF-κB pathway was involved in TNFα-mediated IFNε gene expression, primary cervical cells were treated with the IκB kinase (IKK) inhibitor, BAY 11-0782, to block NF-κB activation. BAY 11-0782 blocked TNFα-mediated IFNε induction, suggesting the role for the NFκB pathway in IFNε gene regulation (Fig. 2a). Note that the suppression of IFNε induction by BAY 11-0782 was not due to cytotoxicity as determined by using the CytoTox-Glo Cytotoxicity assay (data not shown) (Promega). We confirmed that the IKK inhibitor blocked NFκB p65 serine-536 phosphorylation in response to TNFα stimulation (Fig. 2b). We then generated a DNA construct with a luciferase reporter gene under the control of IFNε promoter (nt − 1000 to + 20). The pGL3 with IFNε promoter construct had a low basal luciferase activity compared to the promoterless pGL3 basic vector, and TNFα significantly induced the IFNε promoter activity (Fig. 2c). Importantly, BAY 11-0782 blocked TNFα-mediated activation of the IFNε promoter, suggesting the involvement of the NFκB pathway in IFNε gene regulation (Fig. 2d).

Our results showed that TNFα induced IFNε gene expression in both A2EN and CECs through NF-κB signaling. The degree of induction in these cells was more pronounced than that previously reported in HeLa cells [9]. Additionally, we observed TNFα-mediated IFNα induction, which is absent in HeLa cells [9]. The discrepancy may be due to the limited passage of A2EN and CECs, which could preserve a more primary cell-like immune response. Activation of the IFNε promoter by TNFα was not observed in the study by Matsumiya et al. [9]. The discrepancy may be due to the different promoter region (Matumiya, nt − 497 to + 296 vs nt − 1000 to + 20 in this study) or different cell types. NF-κB binding sites have been identified at the − 53 and − 854 nt position in the proximal promoter of IFNε [2], which were included in our promoter construct. Our study suggests the role for NFκB in IFNε gene regulation. Because both TNF-α and IL-1β activate NF-κB, p38 MAPK, and JNK [14, 15], further analysis of the IFNε promoter function and of the role of transcriptional factors and kinases involved in IFNε gene expression will produce a better understanding of IFNε regulation by cytokines at mucosa.

Although TNFα-mediated induction of IFNα and IFNε was consistent between A2EN and CECs, induction of IFNε in response to various cytokines was noted to be stronger in A2EN cells than CECs. Similarly, suppression of IFNs by IL-22 was apparent in A2EN cells. Differential profiles of IFN regulation between A2EN and CECs may be due to the tissue origin (e.g. ectocervix or endocervix), the presence of HPV E6/E7 in A2EN cells, or cells at different differentiation states. A2EN is an endocervical cell line, representing a uniform cell population, whereas CECs were composed of heterogeneous epithelial cells from cervical tissues, mostly ectocervix. It is possible that some cell populations in CECs may not be responsive to stimulation.

In summary, our study demonstrated that TNFα and IL-1β were potent inducers of IFNε and that the NF-κB pathway was involved in TNFα-mediated IFNε gene regulation. Expression of IFNα, IFNβ and IFNε was regulated differentially in response to specific cytokines, and gene regulation of IFNs by specific cytokines can be concentration-, time-, and cell type-dependent.

## Materials and methods

### Reagents

Keratinocyte Serum Free Media (1×) (KSFM) and all reagents for transfection and reverse transcription were purchased from Thermo Fisher Scientific (Waltham, MA). RPMI-1640 medium and fetal bovine serum (FBS) were purchased from Sigma-Aldrich (St. Louis, MO). Recombinant human IL-17A, IL-22, IL-1β and IL-8 were purchased from Shenandoah Biotechnology (Warwick, PA). Human IL-6 and TNFα were purchased from R&D Systems (Minneapolis, MN). IFNα2a was purchased form PBL assay science (Piscataway, NJ).

### Cell culture

The immortalized epithelial cell line A2EN cells were generated from endocervical tissues of healthy donors as described previously [16]. A2EN cells (passage 19)  were maintained in KSFM supplemented with 5 ng/ml epidermal growth factor, 50 µg/ml bovine pituitary extract and 0.2 mM CaCl_2_ at 37 °C in a 5% CO_2_ humidified incubator. Cells were limited to 5–6 passages. Primary cervical cells were prepared based on published protocols with modification [16]. Briefly, cervical tissues without gross pathology were obtained from women undergoing therapeutic hysterectomy. Human subject protection received IRB approval from Rutgers-New Jersey Medical School. All donors were HIV negative. Cervical tissues were cut into 2 mm^3^ pieces and digested with collagenase IV at 37 °C for 90 min. After passing through a 70 μm cell strainer, the cells were collected by centrifugation and washed with PBS. Isolated cervical cells were cultured overnight at 37 °C in a CO_2_ incubator to allow epithelial cells to attach. Non-attached cells were removed by washing off non-adherent cells extensively. Adherent epithelial cells were cultured until cells reached their confluency. Primary human cervical epithelial cells were maintained in RPMI in the presence of 10% FBS.

Cervical cells were stained with the cervical epithelial marker CK-19 and analyzed by FACS to confirm their purity. Cells less than five passages were used in the experiments.

### Real-time PCR

Total RNA was isolated from cells using TRIzol^®^ (Life Technologies, CA). To synthesize first-strand cDNA, 1000 ng of total RNA, oligo d(T)_16_ (25 μg/ml) and dNTP (0.5 mM) were incubated at 65 °C for 5 min and quick-chilled on ice. RT was performed at 42 °C for 50 min and 70 °C for 15 min using SuperScript III Reverse Transcriptase. The PCR reaction contained cDNA equivalent to 30 ng of RNA input, 200 nM primer sets, and SYBR Green Master Mix (QIAGEN, Valencia, CA), and was run in a StepOnePlus real-time PCR system (Life Technologies, Carlsbad, CA). PCR conditions included 95 °C denaturation for 10 min, 40 cycles of 95 °C for 15 s, 60 °C for 60 s. PCR products were quantified and normalized relative to the amount of GAPDH cDNA products as described by Schmittgen et al. [17]. Relative quantification of gene expression was calculated using the ΔΔCt (Ct, threshold cycle of real-time PCR) method according to the formula: $\Delta {\text{CT}} = {\text{Ct}}_{\text{GAPDH}} {-}{\text{Ct}}_{\text{target}} , \, \Delta \Delta {\text{Ct}} = \Delta {\text{Ct}}_{\text{control}} {-} \, \Delta {\text{Ct}}_{\text{IFN}} ,{\text{ Ratio}} = 2^{{ - \Delta \Delta {\text{Ct}}}} .$ Primer sequences were: IFNε forward (5′-AGC ACT CAT GGG ACT GGA ACT GGA AG-3′), reverse (5′-CAG GTG CTG TAG TCC TGG TT-3′); IFNα forward (5′-CAC ACA GGC TTC CAG GCA TTC-3′), reverse (5′-TCT TCA GCA CAA AGG ACT CAT CTG-3′), IFNβ forward (5′-GAG CTA CAA CTT GCT TGG ATT CC-3′) reverse (5′-CAA GCC TCC CAT TCA ATT CC-3′); GAPDH forward (5′-GCA CCA CCA ACT GCT TAG CAC-3′), reverse (5′-TCT TCT GGG TGG CAG TGA TG-3′).

### Promoter construct and transfection

The IFNε promoter DNA that comprised the 5′-flanking region nt − 1000 to + 20 of the IFNε gene was synthesized and cloned in the Promega pGL3 luciferase reporter basic vector (no promoter) by Genescript. The DNA sequence of the construct was confirmed. To assess the IFNε promoter activity, the pGL3 or pGL3 with IFNε promoter region was transfected into A2EN (0.125μg plasmid DNA) by Lipofectamine 2000 and incubated for 24 h. Cells were pre-treated with or without the IKK inhibitor BAY-11-7082 for 1 h before stimulation with TNFα at 1 ng/ml. Cells were cultured for 24 h before lysis in passive lysis buffer (Promega, Madison WI). Luciferase activity [in relative light units (RLUs)] was measured on a 2300 EnSpire Multimode Plate Reader (PerkinElmer, Waltham, MA).

#### Statistical analysis

Statistical comparisons were performed by using two-tailed paired-Samples t test (GraphPad Prism 5.01, LaJolla, CA). A *p* value of < 0.05 was considered significant.

## Abbreviations

IFN: interferon
CECs: cervical epithelial cells
ELISA: enzyme-linked immunosorbent assay
h: hour
TNF-α: tumor necrosis factor
IL-1β: interleukin-1β
NF-κB: nuclear factor kappa-light-chain-enhancer of activated B cells
MAPK: mitogen-activated protein kinase
JNK: c-Jun-N-terminal kinase
